# Benefit from decline: the primary transcriptome of *Alteromonas macleodii* str. Te101 during *Trichodesmium* demise

**DOI:** 10.1038/s41396-017-0034-4

**Published:** 2018-01-15

**Authors:** Shengwei Hou, Mario López-Pérez, Ulrike Pfreundt, Natalia Belkin, Kurt Stüber, Bruno Huettel, Richard Reinhardt, Ilana Berman-Frank, Francisco Rodriguez-Valera, Wolfgang R. Hess

**Affiliations:** 1 Faculty of Biology, University of Freiburg, Schänzlestr. 1, D-79104 Freiburg, Germany; 2 Evolutionary Genomics Group, División de Microbiología, Universidad Miguel Hernández, Apartado 18, San Juan, 03550 Alicante Spain; 3 Mina and Everard Goodman Faculty of Life Sciences, Bar-Ilan University, Ramat Gan, 52900 Israel; 4 Max Planck-Genome-Centre Cologne, Carl-von-Linné-Weg 10, D-50829 Köln, Germany; 5 Freiburg Institute for Advanced Studies, University of Freiburg, Albertstr. 19, D-79104 Freiburg, Germany; 6 Present Address: ETH Zürich, Department of Civil, Environmental and Geomatic Engineering, Institute of Environmental Engineering, Stefano-Franscini-Platz 5, CH-8093 Zürich, Switzerland

**Keywords:** Water microbiology, Microbial ecology, Bacterial genetics, Marine microbiology

## Abstract

Interactions between co-existing microorganisms deeply affect the physiology of the involved organisms and, ultimately, the function of the ecosystem as a whole. Copiotrophic *Alteromonas* are marine gammaproteobacteria that thrive during the late stages of phytoplankton blooms in the marine environment and in laboratory co-cultures with cyanobacteria such as *Trichodesmium*. The response of this heterotroph to the sometimes rapid and transient changes in nutrient supply when the phototroph crashes is not well understood. Here, we isolated and sequenced the strain *Alteromonas macleodii* str. Te101 from a laboratory culture of *Trichodesmium erythraeum* IMS101, yielding a chromosome of 4.63 Mb and a single plasmid of 237 kb. Increasing salinities to ≥43 ppt inhibited the growth of *Trichodesmium* but stimulated growth of the associated *Alteromonas*. We characterized the transcriptomic responses of both microorganisms and identified the complement of active transcriptional start sites in *Alteromonas* at single-nucleotide resolution. In replicate cultures, a similar set of genes became activated in *Alteromonas* when growth rates of *Trichodesmium* declined and mortality was high. The parallel activation of *fliA, rpoS* and of flagellar assembly and growth-related genes indicated that *Alteromonas* might have increased cell motility, growth, and multiple biosynthetic activities. Genes with the highest expression in the data set were three small RNAs (Aln1a-c) that were identified as analogs of the small RNAs CsrB-C in *E*. *coli* or RsmX-Z in pathogenic bacteria. Together with the carbon storage protein A (CsrA) homolog Te101_05290, these RNAs likely control the expression of numerous genes in responding to changes in the environment.

## Introduction

*Trichodesmium* is a marine cyanobacterium greatly impacting the biogeochemical cycles of carbon and nitrogen in the oligotrophic subtropical and tropical oceans [1]. *Trichodesmium* forms expansive blooms at the surface of the ocean that are easily tracked by satellites [2]. When these blooms decay, large amounts of nutrients are released into the sea [1, 3]. Recent DNA-based research into the microbial community associated with *Trichodesmium* suggested complex metabolic interactions and far-reaching ecological implications [4–7], which called for detailed studies of how these microbial associations work. The nearly ubiquitous presence of *Alteromonas* in natural *Trichodesmium* samples [8] and their multifaceted capabilities of recycling marine dissolved organic carbon [9] point to the scientific value of understanding this particular interaction in more detail. Focusing on the phytoplankton physiology, the effects of an *Alteromonas* co-culture with phytoplankton including *Prochlorococcus* [10–12] and the diatom *Phaeodactylum tricornutum* [13] were studied. Total gene expression has been studied also in environmental *Trichodesmium* blooms [14], but there is no transcriptome study specifically targeting the heterotroph in these interactions.

Heterotrophic gammaproteobacteria that typically occur at a rather low abundance in oligotrophic marine or freshwater habitats [15] can thrive under eutrophic, nutrient-enriched, conditions [16]. When organic material of marine origin was incubated on-board ships or in mesocosms, dense populations of gammaproteobacteria such as *Alteromonas* were observed repeatedly [17–19]. *Alteromonas* have large cells and genomes and can grow rapidly when nutrients are abundant [20]. In offshore oligotrophic pelagic habitats and in most oligotrophic tracts of the central ocean gyres, the transient nutrients discharged from particulate organic matter provide opportunities for the swift growth of *Alteromonas* [21–23]. In fact, growing evidence indicates that the oligotrophic ocean is a heterogeneous environment with tiny, transient nutrient patches appearing regularly, for example, from fecal pellets or sinking dead phytoplankton, thus providing nutrient gradients at the microscale. These “hot-spots” favor motile copiotrophic bacteria such as *Alteromonas* [24–26]. Although the available *Alteromonas* genomes do not recruit a large number of reads from most metagenomes [27–29], metatranscriptomic analyses have supported *Alteromonas* as an active constituent of the ocean microbial community with high transcriptional activities despite its low representation in metagenomes [17, 30, 31].

Most published *Alteromonas* genomes demonstrate relatively conserved synteny over their core, which allows the precise identification of variable regions, such as flexible genomic islands (fGIs) [32]. In comparison, *Alteromonas* transcriptomes are poorly characterized. One study investigated the responses of *Alteromonas* to different environmental parameters using two closely related deep-sea ecotype strains isolated from the same environmental sample [33]. Another transcriptomic analysis showed that the co-cultivation of *Alteromonas macleodii* MIT1002 with *Prochlorococcus* significantly impacted the physiology of the latter [34]. However, neither the impact of the associated cyanobacterium on *Alteromonas* nor the gene expression in an expanding population co-existing with a dense primary producer has been elucidated to date.

Here, we isolated and sequenced *Alteromonas macleodii* strain Te101 (from here: *Alteromonas* Te101) from a culture of *Trichodesmium erythraeum* IMS101 (from here: *Trichodesmium* IMS101), with which it had been co-existing for years. Raising the salinity from 37 to 43 parts per thousand (ppt) in these cultures inhibited the growth of *Trichodesmium* but stimulated the growth of *Alteromonas*, which tolerates up to 180 ppt salinity [28]. We isolated the total RNA from biological replicate cultures at 43 ppt salinity and parallel cultures with healthy *Trichodesmium* IMS101 (37 ppt). Using differential RNA sequencing (dRNA-Seq) [35], we inferred the complement of active transcription start sites (TSSs) of *Alteromonas* Te101 at single-nucleotide resolution. The analysis of differentially expressed genes revealed the activation of a carbon storage regulator A and B-C (CsrA-CsrB-C)-like regulatory mechanism at the heart of the response to *Trichodesmium* decay.

## Materials and methods

### Culturing conditions, RNA preparation, and transcriptome sequencing

Non-axenic *Trichodesmium* IMS101 cultures were grown in YBCII [36] at 25 °C under a 12:12 light–dark cycle at ~80 μmol photons m^−2^ s^−1^ white light. During exponential growth, biomass of associated bacteria (predominantly *Alteromonas*) is generally negligible compared with the cyanobacterial biomass. However, stress conditions leading to high mortality of *Trichodesmium* enhanced the growth of associated bacteria. Subsamples were collected for chlorophyll *a* extraction by boiling in 90% methanol and spectrophotometric analysis at 664 nm [37] every 1–3 days. On day 9, the extracts were scanned for pigment absorbance between 520 and 760 nm using a Cary 300 (Agilent Technologies) spectrophotometer. For transcriptome analysis, two replicate batch cultures were grown at 37 ppt (normal YBCII) or 43 ppt (increased salt concentration by adding NaCl to 736 mM) for 9 days in 1 L Pyrex bottles with air bubbling and harvested in the middle of the light phase.

Two replicate dRNA-Seq complementary DNA (cDNA) libraries were constructed for each of the two conditions, yielding four 5’P-dependent Terminator Exonuclease (TEX) processed dRNA-Seq libraries. For each dRNA-Seq library, a minus library was prepared from the same TEX-processed sample, which was not further treated with tobacco acid pyrophosphatase before 5' linker ligation, resulting in sequencing the left-over processed transcripts. In order to cover the full length of the transcripts, one additional cDNA library was constructed by pooling aliquots from the four samples for classical RNA-Seq analysis in which the TEX treatment was omitted (RNA-Seq library). Single-end transcriptome sequencing was performed on the Illumina HiSeq 2000 sequencing system with a read length of 100 nt. After adapter removal and quality trimming, >40 million and 18 million reads were generated for each of the dRNA-Seq and minus libraries, separately. Details of RNA isolation and library preparation were presented previously [38, 39].

### Strain isolation and genomic sequencing

*Alteromonas* Te101 was isolated from *Trichodesmium* IMS101 cultures by dilution and plating on marine agar (12% sea salt (Sigma), 0.5% peptone, 0.1% yeast extract, and 1.5% agar). Single colonies were then grown in liquid culture (12% sea salt (Sigma), 0.5% peptone, and 0.1% yeast extract) at 25°C. Genomic DNA for Single Molecule Real Time (SMRT) sequencing analysis was extracted from 80 mL cultures collected by centrifugation. The pellet was re-suspended in 1 mL SET on ice (25% (w/v) sucrose, 1 mM EDTA, and 50 mM Tris, pH 7.5). One-fourth volume of 0.5 M EDTA, 2% sodium dodecyl sulfate, and 1.5 mg proteinase K (Sigma) were added for cell lysis at 50 °C overnight. Following phenol/chloroform extraction, one volume of 2-propanol (Roth, Germany) was added for DNA precipitation at room temperature for 30 min. The precipitate was washed once in H_2_O/2-propanol (1:1) and once in 2-propanol, followed by 10 min centrifugation at 10,000 g at 4 °C. The pellet was washed with 70% EtOH, dried for 10 min, and re-suspended in 100 µL H_2_O. One µL of RNase A (Sigma) was added and the tube was incubated at 37 °C with shaking at 260 rpm overnight. RNase was removed by another round of phenol extraction and precipitation. The DNA was re-suspended in 75 µL H_2_O and quantified using Quant-iT® PicoGreen® dsDNA Reagent (Invitrogen). DNA libraries were prepared according to the large SMRTbell gDNA protocol (Pacific Biosciences) with a 10-kb insert size. Genomic DNA was sequenced with a PacBio RS II platform. Generated reads were trimmed and de novo assembled using the HGAP workflow version 2 (Pacific Biosciences). Genomes were annotated using PGAAP (http://www.ncbi.nlm.nih.gov/genome/annotation_prok/).

### Bioinformatic methods

fGIs were identified as regions where synteny (≥10 kb) was broken between genomes of different isolates [40]. To detect these genomic islands and to assess genome rearrangements, reciprocal BLASTN and TBLASTX searches between the *Alteromonas* Te101 and previously assembled *Alteromonas* genomes [28] were performed. The Average Nucleotide Identity (ANI) between strains was calculated using the JSpecies software v1.2.1 with default parameters [41]. The COG database [42] was used to identify conserved proteins requiring an e-value < 1e^−5^, >80% query coverage, and >30% identity. Concatenated proteins were aligned with Kalign [43], and a maximum likelihood tree was generated by FastTree [44] using a JTT + CAT model and gamma approximation.

For transcriptome analysis, we followed the workflow for read cleaning and quality control described by [35]. Reads from all libraries were aligned to the *Alteromonas* Te101 genome at 99% identity using segemehl v0.2.0 [45]. For TSS identification, a replicate-assisted background subtraction algorithm was designed and applied. A TSS located within 200 nt upstream of an annotated protein-coding gene or giving rise to reads overlapping such a gene was classified as a gene TSS (gTSS). A TSS located within an annotated gene or antisense to it (plus 50 nt up or downstream) was classified as an iTSS or aTSS, respectively. A TSS located in an intergenic region or upstream of a non-coding (nc)RNA, including small RNA (sRNA), ribosomal RNA (rRNA), and transfer RNA (tRNA) genes, was designated a non-coding TSS (nTSS). The implementation of this algorithm can be found at https://github.com/housw/GRPutils/blob/master/tss_analysis_pipline.sh.

For differential expression analysis, the TSS counts were normalized using the Trimmed Mean of M-values method in edgeR v3.14.0 [46]. Dispersions were estimated by treating samples between 37 and 43 ppt as replicates using the quantile-adjusted conditional maximum likelihood method, and TSSs that were differentially expressed between 43 and 37 ppt were called by the exactTest function in edgeR with an adjusted *p*-value cutoff of 0.05.

Potential CsrA-binding sites were searched with the regular expression “GGA[ACGT]{4,70}GGA[ACGT]{2,12}$” [47] and additionally by using the CSRA_TARGET algorithm [48]. To detect potential promoter motifs, sequences 200 nt upstream of the TSSs were scanned by XXmotif [49]. A more detailed version of the bioinformatics analyses can be found in the Supplementary Information.

### Accession information

*A. macleodii* Te101 has been deposited in the DSMZ culture collection (no. DSM 104636). PacBio raw reads, genome sequences, and annotations have been deposited at NCBI under BioProject ID PRJNA355640. Primary transcriptomic reads can be found at NCBI Sequence Read Archive under BioProject ID PRJNA237745.

## Results and Discussion

### Microbial community associated with *Trichodesmium* at different salinities

**Fig. 1:**
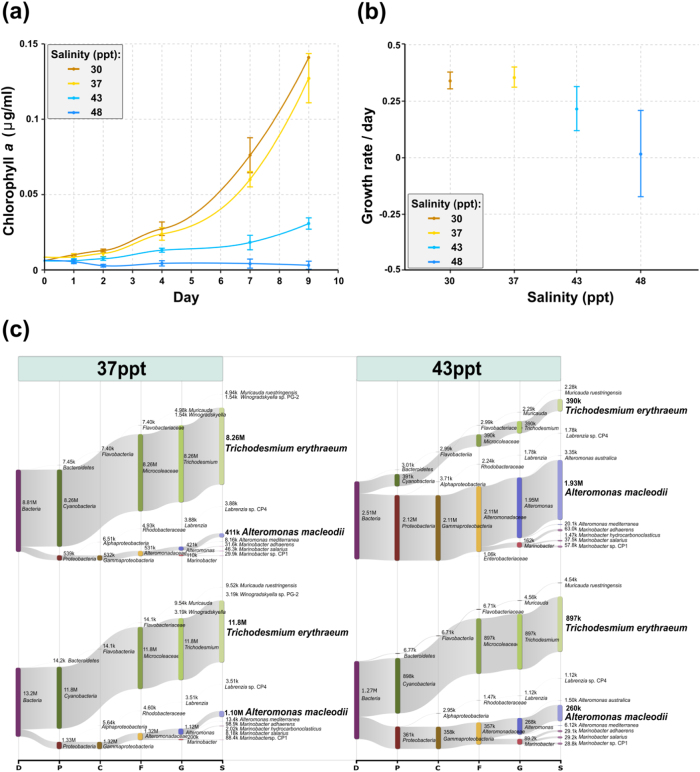
Chlorophyll *a* concentration **a** and cell growth rates **b** of *Trichodesmium* cultures at different salinities. The error bars represent standard errors in **a** and standard deviations in **b**. Only exponential growth phase (days 2–9) was considered in calculating the cell growth rates in **b**. **c** Hierarchical taxonomy assignment of metatranscriptome reads. Non-rRNA reads of dRNA-Seq libraries were used for taxonomy assignment with Centrifuge [105]. Taxa were aligned hierarchically from domains to species. The number of assigned reads was shown on top or next to each taxon. Only taxa recruiting >1000 reads are shown. D domain, P phylum, C class, F family, G genus, S species, M million, K thousand

Non-axenic *Trichodesmium* IMS101 cultures were grown in YBCII media for 9 days at salinities of 30, 37, 43, and 48 ppt, in two replicates for each treatment. *Trichodesmium* grew exponentially at the lower salinities (30 and 37 ppt) that represent the salinity range from estuarine to oceanic waters with an average salinity of 35 ppt in pelagic surface oceans [50, 51] (Figs. 1a, b). Growth rates declined, although biomass still increased, when ambient salinity was increased to 43 ppt (Figs. 1a, b) [38], while growth was severely inhibited at 48 ppt salinity, as shown by the spectral scan of pigments (Figure S1). Although *Trichodesmium* also thrives in high salinity waters such as in the Gulf of Aqaba (Red Sea; salinity ~40.8) [52, 53], our data show that salinities ≥43 ppt induce great stress, declining growth rates, and enhanced mortality.

This increase in salinity affected the composition of the microbial community, as shown here by the hierarchical taxonomic classification of transcriptome reads (Fig. 1c). At 37 ppt salinity, ~90% of assignable reads were classified as *Trichodesmium erythraeum* in both replicates, 4.6% and 8.4% of assignable reads were classified as *Alteromonas* and a minor number of reads were classified as *Marinobacter*. In contrast, at 43 ppt at the same day, the assignable read share of *Trichodesmium* was 14.1% in replicate 1, whereas the share of *Alteromonas* and *Marinobacter* increased to 70.6% and 5.9%, respectively. In replicate 2, the relative abundance of *Trichodesmium* was also lower (65.4%), and the share of *Alteromonas* (19.5%) and *Marinobacter* (6.5%) higher, although less pronounced than in replicate 1. Hence, although developing with different dynamics, the expanding *Alteromonas* Te101 populations in both replicates provided the opportunity to investigate its transcriptome composition in the context of stressed *Trichodesmium*.

### Comparative genomic features of *Alteromonas* Te101

We are unaware of an axenic isolate of *Trichodesmium* IMS101 and the presence of co-occurring gammaproteobacteria is well known [8]. Through multiple dilution and streak cultivation, we segregated an axenic *Alteromonas* strain from a non-axenic culture of *Trichodesmium* IMS101. Based on its 16S rRNA sequence, the strain was classified as belonging to the species *A. macleodii*. The strain isolation was repeated from the continuously propagated cultures after 1 year and 16S sequencing confirmed the previously identified *Alteromonas*.

**Fig. 2:**
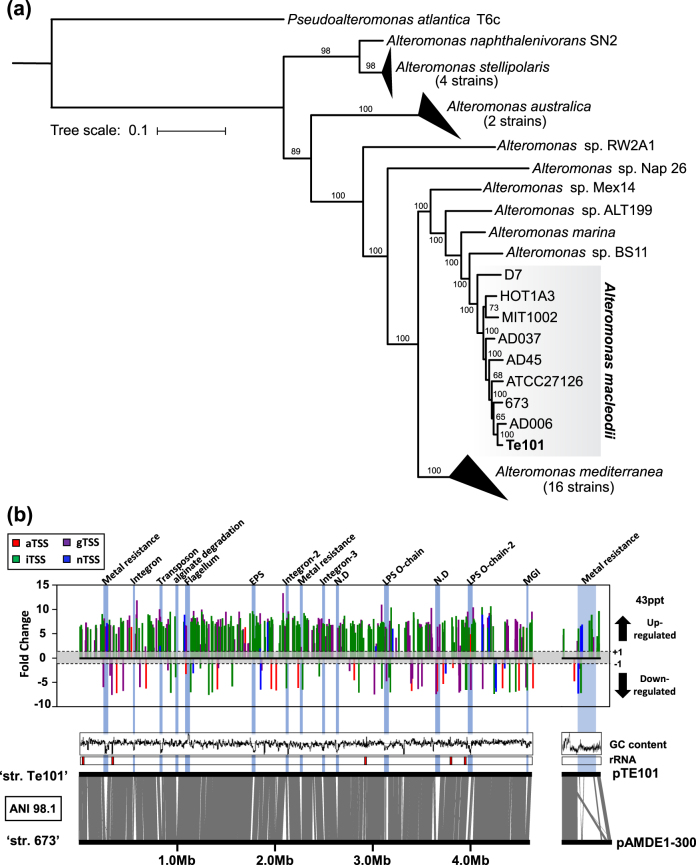
**a** Phylogenetic relationships between *Alteromonas* Te101 and other members of the genus *Alteromonas*. The tree is based on a concatenated set of 1015 proteins from the shared core proteome of all available *Alteromonas* genomes and *Pseudoalteromonas atlantica* T6c, which was used as an outgroup. The numbers at branches represent bootstrap values in percent. **b** Flexible genomic islands (fGIs) and differentially expressed TSSs. Differentially expressed TSSs are visualized on the upper panel, the *y* axis shows the log_2_-transformed fold changes. Genomic-wide synteny and fGIs (vertical blue rectangles) are shown on the lower panel. The chromosome of the *A. macleodii* ‘English Channel 673’ was used as a reference to detect fGIs in the *Alteromonas* Te101 chromosome. In the same way, the plasmid pTE101 was compared against the plasmid pAMDE1-300 of *A. mediterranea* strain DE1. If known, the fGI’s function is given on top. N.D represents not determined. For the list of genes within these fGIs, see Table S1

Using SMRT sequencing, complete sequences of the 4.63 Mb chromosome and a single plasmid of 237 kb, named pTE101, were recovered (Table 1). A phylogenomic tree inferred from 1015 concatenated core proteins (Fig. 2a) confirmed the initial 16S-based phylogeny of *Alteromonas* Te101 and demonstrated its clustering with other *A. macleodii* strains, sharing an ANI > 95%. We used strain ‘English Channel 673’ (ANI 98.1%) for comparison because its genome is complete and well annotated [28].

**Table 1:** Genome information for *Alteromonas* Te101 and composition of the primary transcriptome for the two tested conditions

*Alteromonas* Te101	Chromosome	Plasmid
Genome size (bp)	4,630,082	237,311
Scaffolds	1	1
GC content (%)	44.7	41.7
Coding density (%)	88	85
Median intergenic distance (bp)	94	66
CDS	3,920	239
tRNA	72	0
rRNA	16	0
Total number of TSS	2,181	99
Number of gTSS (total %)	1,282 (58.78)	33 (33.33)
Number of aTSS (total %)	130 (5.96)	16 (16.16)
Number of iTSS (total %)	595 (27.28)	35 (35.35)
Number of nTSS (total %)	174 (7.98)	15 (15.15)
TSS detected at 37 ppt condition	1,365	66
Number of gTSS (total %)	892 (65.35)	27 (40.91)
Number of aTSS (total %)	99 (7.25)	11 (16.67)
Number of iTSS (total %)	225 (16.48)	19 (28.79)
Number of nTSS (total %)	149 (10.92)	9 (13.64)
TSS detected at 43 ppt condition	1,928	80
Number of gTSS (total %)	1,179 (61.15)	29 (36.25)
Number of aTSS (total %)	80 (4.15)	11 (13.75)
Number of iTSS (total %)	515 (26.71)	28 (35.00)
Number of nTSS (total %)	154 (7.99)	12 (15.00)

Owing to the well-preserved synteny among the members of this genus, we detected 14 fGIs in the chromosome harboring ca. 8% (298/3920) of all genes (Fig. 2b, Table S1). These fGIs contain clusters of flexible genes that occupy equivalent genomic locations in different *Alteromonas* strains [54]. Two major classes of fGIs have been defined: additive fGIs, which are recombination hotspots for the integration of various different alien genes, and replacement fGIs, which also contain different but functionally related genes for outer layer glycoproteins or polysaccharides [32]. Four replacement fGIs were previously described in *Alteromonas* [32], and *A. macleodii* 673 and *Alteromonas* Te101 have different versions of three of them. However, both strains share a nearly identical flagellum glycosylation island, despite the temporal and geographical distances between the isolates (Fig. 2b). In both strains, the additive fGIs contain long gene cassettes, such as integrons and mobilizable genomic islands and mobile genetic elements (transposons), or are related to specific metabolic features, such as alginate degradation and metal resistance (Fig. 2b).

Comparison of the pTE101 plasmid sequence revealed a region of ~140 kb encoding the machinery for conjugal transfer and plasmid maintenance that is highly syntenic with *A. macleodii* 673 plasmid pAMDE1-300 (Fig. 2b). In the flexible region, both plasmids contain multiple insertion sequence elements and gene clusters involved in heavy metal resistance, whereas a previously identified hybrid NRPS-PKS cluster [55] is restricted to pAMDE1-300.

### The primary transcriptome architecture of *Alteromonas* Te101 differs from other bacteria

We identified 2280 TSSs for the *Alteromonas* Te101 chromosome and plasmid pTe101 active at least at one condition. Of these, 57.7% were gTSSs, 27.6% iTSSs, 8.3% nTSSs, and 6.4% aTSSs (for an explanation of the different types of TSSs please refer to Bioinformatic methods section); a total of 2008 were active at 43 ppt, and 1431 at 37 ppt salinity (Table 1). The median 5′-untranslated region (UTR) length was 63 nt, but we also found 10 leaderless transcripts and 34 long 5′-UTRs (>200 nt), pointing to different modi of post-transcriptional regulation (Figure S2, Table S2). The 1315 gTSSs were associated with 1191 annotated genes, of which 108 had more than one gTSS, indicating more complex transcriptional regulation. Although the different percentages of predicted TSS types should be considered with caution since there are differences in the growth conditions or the definition of transcript types, the share of gTSSs was considerably higher in *Alteromonas* Te101 (Figure S3) than in other bacteria and archaea, for example, 30% in the co-cultured *Trichodesmium* IMS101 [39], 50.4% in *Xanthomonas campestris* B100 [56] and 17.3% in *Prochlorococcus* sp. MIT9313 [57]. Conversely, high numbers of aTSSs have been reported in several bacteria, including *Trichodesmium* [39, 53, 56, 58–62], whereas only ~6% of all TSSs in *Alteromonas* Te101 were classified as aTSS (Table 1). These results suggest differences in the transcriptome architecture of *Alteromonas* Te101 compared with other bacteria (Figure S3). The genome-wide visualization of identified TSSs and transcriptome coverage is shown in Supplementary Dataset 1.

### Multiple regulators are involved in the lifestyle decision to activate flagellum biosynthesis and motility

Principal component analyses revealed that the *Alteromonas* transcriptomes were qualitatively highly similar at 43 ppt salinity in both replicates compared with the transcriptomes at 37 ppt (Figure S4). The divergence between the replicates at 37 ppt might be due to low sequencing depth, which can be sensitive to variations of culture conditions. In addition, the different growth stages of *Alteromonas* cannot be excluded, which might be affected by uncontrolled environmental and uncharacterized biological factors. Nevertheless, similar transcriptional activities were observed at 43 ppt, which allows us to determine differentially expressed genes robustly. Thus, the replicates were fed into differential expression analysis to identify the TSSs that were up or downregulated at 43 ppt. The list of TSSs and their expression levels under both conditions, fold changes, and a global overview of their distribution along the chromosome are given in Figure S5, Table S3 and Supplementary Dataset 1.

Benefiting from the precise identification of TSS, our primary transcriptomic data makes it possible to compare promoter activities across different conditions. Compared with the classical RNA-Seq based quantification, TSS-based quantitative analysis provides a higher resolution for genes with multiple TSSs, and for genes with abundant internal transcripts. Applying a log_2_-transformed fold change (logFC) ≥ 1 and a false discovery rate <0.05, we identified 504 and 56 significantly up or downregulated genes, respectively (Tables S4 and S5). Although 43 ppt does not appear to induce conspicuous stress in *Alteromonas*, consistent with the reported stability of *Alteromonas* up to a salinity of 180 ppt [28], we analyzed the salt-specific responses in this study. The basic mechanism to deal with high osmolarity is the accumulation of compatible solutes including amino acids and derivatives such as glycine betaine, proline, and ectoine. These pathways were not differentially expressed (sodium/proline symporter TE101_09200, TE101_06005; choline transporter TE101_00375, TE101_14030, TE101_16255, TE101_08845) (Table S3), indicating that *Alteromonas* could deal with the tested salinity differences without transcriptional changes for these genes. However, we cannot exclude the possibility that *Alteromonas* Te101 benefitted from the uptake of large quantities of homoserine betaine, which is the major compatible solute of *Trichodesmium* IMS101 [38]. Our results showed that for *Alteromonas*, one homeostatic mechanism in response to a slight variation in salinity was the activation of K^+^ and Na^+^ transporters (TE101_03025, TE101_06605).

**Fig. 3:**
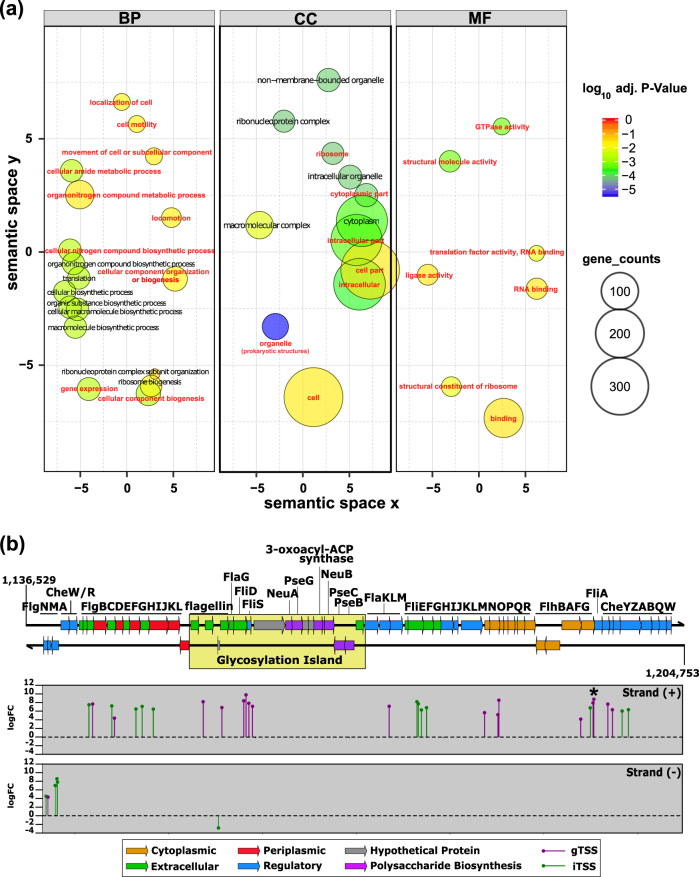
**a** Enriched GO terms for genes upregulated at 43 ppt. In order to cluster semantically similar GO terms together and to reduce redundancy, pairwise semantic similarities of enriched GO terms were calculated using REVIGO with the SimRel method [106]. Terms with a dispensability <0.4 were taken as representatives and highlighted in bold font and red color, redundant terms are shown in black. Only GO terms with an adjusted *p*-value < 0.05 are shown. Circle sizes correspond to the counts of differentially expressed genes involved in that GO term. BP biological process, CC cellular component, MF molecular function. **b** Expression of the flagellar gene cluster. TSSs differentially expressed at 43 ppt are indicated in the lower two panels, separately for the forward and the reverse strand. Two strongly activated TSSs for the *fliA* gene are highlighted with an asterisk symbol. The variable region is highlighted by the yellow box. logFC log_2_-transformed fold changes

Among several terms, Gene Ontology (GO) analysis revealed “localization of cell” and “movement of cell or subcellular component” as enriched terms within the *biological process* domain for TSSs upregulated at 43 ppt (Fig. 3a and Table S6). Consistent with this classification, 34 upregulated TSSs were found within the motility gene cluster alone. Two of these serve the regulator FliA (sigma 28, gene TE101_05165), which allows the expression of genes belonging to the late operon and genes encoding components of the flagellar filament, such as the flagellar hook protein (FliD; TE101_04980), the flagellar export chaperone FliS (TE101_04985), or flagellins (FliC; TE101_04960 and TE101_04970). Several upregulated genes encode proteins involved in sensing and responding to environmental cues (Table S7). Only a single iTSS, possibly a post-transcriptional regulator located in the flagellum glycosylation fGI, was downregulated (Fig. 3b). These data clearly showed that flagellar filament synthesis was stimulated at 43 ppt. This observation is consistent with a report using quantitative video microscopy of bacteria swarming around a lysing diatom cell and a resource utilization model, which revealed that motile, chemotactic copiotrophs have a competitive advantage over non-motile cells when a phytoplankton bloom collapses [25].

In addition to the sigma factor FliA, we found four more upregulated sigma factors: RpoS (sigma S, Te101_02645), RpoN (sigma 54; TE101_05050), RpoH (sigma 32; TE101_17790), and RpoE (sigma 24; TE101_13105). The homologs of the latter three in *Escherichia coli* (*E. coli*) are involved in nitrogen metabolism regulation, and the responses to heat shock and cell surface stress [63]. RpoS (74% identity with *E. coli* RpoS) [64] was, with a logFC of 11.6 (iTSS-578989), the second-most strongly induced gene at 43 ppt (Table S4). This finding implied that RpoS in *Alteromonas* Te101 was one of the main regulators during the tested growth condition. In *E.coli*, RpoS is important for survival during nutrient deprivation or the stationary phase of growth [65]. In a broader set of bacteria, RpoS is often considered as a general stress response factor because it governs the expression of multiple genes that mediate physiological changes related to osmotic, pH, oxidative, and heat stresses, and the production of virulence factors. In the marine bacterium *Vibrio cholera*, the *rpoS* knockout mutant was deficient in its capacity to produce or secrete an extracellular protease [66]. An *rpoS*-deficient mutant of *Salmonella enterica* serovar Typhimurium showed significant attenuation in virulence [67]. Furthermore, a previous RNA-seq study in *A. mediterranea* where the transcriptional response to different growth conditions (including starvation) was investigated showed that *rpoS* was not differentially expressed when comparing starvation with growth in rich medium [33]. These data suggest that in *Alteromonas*, and probably other opportunistic microbes, RpoS is involved in acclimation responses to changing environments as it is one of the most critical stress acclimation factors and not only in nutrient deprivation conditions [65, 68]. Moreover, RpoS in *E. coli* is associated with biofilm formation [69], and the repression of flagellar gene transcription during the exponential growth phase [70, 71]. However, the here observed parallel activation of *fliA* and *rpoS* suggests the stimulation of multiple physiological processes, including motility, acclimation to higher salinity, and likely the assimilation of *Trichodesmium*-released nutrients. Consistent with the activated expression of these alternative sigma factors, the transcription of the vegetative sigma factor 70 (TE101_03190) decreased. These results suggest that the activation of flagellum biosynthesis and motility is under the control of multiple regulators and part of a complex lifestyle decision.

In the *Trichodesmium* primary transcriptome, increased expression of homoserine methyltransferase (Tery_2447) was observed at 43 ppt salinity (Table S8), consistent with its function in the synthesis of the major compatible solute homeserine betaine [38]. In contrast, the impaired growth of *Trichodesmium* at higher salinities (Figs. 1a, b) correlated with the downregulation of genes involved in major cellular component and compound biogenesis or photosynthesis (Table S8, S9).

### Implications for the marine nitrogen and carbon cycles

*Alteromonas* are copiotrophic bacteria that are commonly found in oceanic phytoplankton bloom and mesocosm studies [17, 20, 72–74]. The physiological and metabolic activities of *Alteromonas* affect the interacting organisms and contribute to the biogeochemical cycling of carbon and nitrogen in the marine environment [21, 22, 24, 25]. Our transcriptomic data allow detailed insight into the genetic underpinnings of how *Alteromonas* metabolism changes upon phytoplankton decay.

**Fig. 4:**
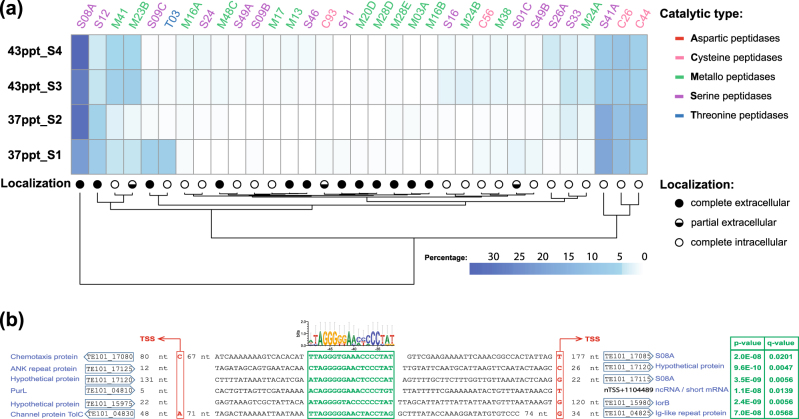
**a** Expression of peptidases in *Alteromonas* Te101. For each MEROPS subfamily, gTSS counts were summed and the percentage from the total peptidase-associated gTSS count from all four samples was used for visualization. Only subfamilies with a column sum ≥ 1% are shown. Columns were clustered using complete linkage hierarchical clustering based on Euclidean distances. Predicted localizations of peptidase subfamilies are given at the ends of the branches. **b** The promoter regions of two S08A extracellular peptidase genes, of *iorB* (TE101_15980) encoding isoquinoline 1-oxidoreductase, of TE101_17120 encoding a hypothetical protein and of nTSS + 1104489, from which an sRNA or a short mRNA is transcribed, share the presence of a palindromic sequence element (boxed in green) at a conserved distance to the TSS (boxed in red). The *p*-value and *q*-value columns show the probabilities and false discovery rates of finding these motifs against the whole genome

Some of the marine MG-II/III Euryarchaeota are capable of utilizing extracellular proteins and peptides for rapid heterotrophic growth [75–77]. Among bacteria, *Alteromonas* from the Arctic Chukchi Sea was identified to have major proteolytic activities [78]. Based on previously established methods [35, 75], we identified 154 genes encoding peptidases in *Alteromonas* Te101, which belong to 67 MEROPS peptidase subfamilies (Table S10). The six genes encoding extracellular S08A peptidases were the most actively transcribed peptidase subfamily members at both salinities (Fig. 4a; Tables S3 and S10). We identified an 18 nt imperfect palindrome at a conserved position around 30–50 nt upstream of the gTSS of two S08A family peptidase genes and of seven other genes (Fig. 4b, motif 6 in Table S11), indicative of potential concerted regulation of these genes by the same regulator. Other upregulated proteases included the ATP-dependent ClpA (TE101_08955, with the highest logFC of 13.1, Table S4), ClpX (TE101_13295), and zinc metalloprotease TE101_09120. These highly expressed proteases degrade misfolded proteins but can also degrade regulatory proteins [79] or imported extracellular peptides. These data indicate that *Alteromonas* expressed a suite of extra- and intracellular peptidases for the utilization of external proteins at both salinities, consistent with observations that proteolysis provides the major source of amino acids for rapid de novo protein synthesis [80].

*Alteromonas* can utilize a wide range of organic carbon sources, including glucose [81] and complex polymeric compounds such as alginate, agarose, and other polysaccharides [82, 83]. Thus, *Alteromonas* could utilize the carbohydrates released from *Trichodesmium* such as transparent exopolymeric particles [84, 85]. Analysis of the expression level of carbohydrate active enzymes (CAZy) showed that glycosyltransferase family 2 (GT2) genes were actively transcribed in both salinities, whereas carbohydrate esterase family 11 (CE11) and glycosyltransferase family 47 (GT47) exhibited strongly increased transcription at 43 ppt (Table S12).

### A CsrA-B-C-like system in *Alteromonas*

**Fig. 5:**
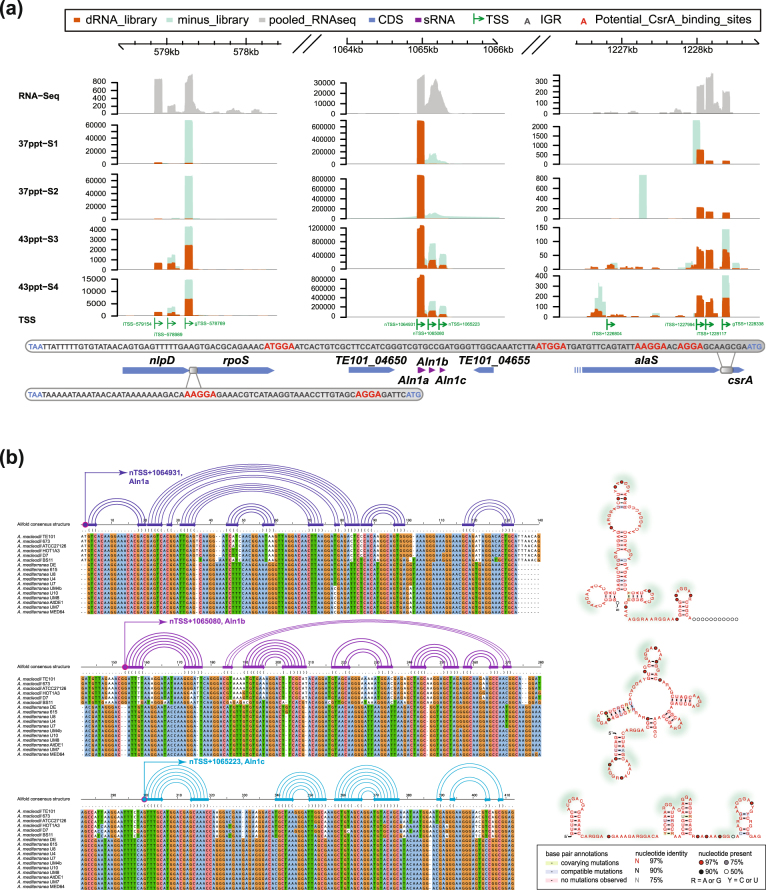
**a** Promoters and read coverage of *rpoS*, Aln1a-c, and *csrA* genes at 37 and 43 ppt. The potential CsrA-binding sites within the *rpoS* and *csrA* 5′-UTRs are highlighted in red. The three sRNAs (Aln1a-c) originate from the 404 nt intergenic region between the genes Te101_04650 and Te101_04655 from three distinct nTSSs. **b** Multiple sequence alignment and predicted secondary structures of Aln1a-c. Identified TSSs are indicated by curved arrows on top of each sequence block. Conserved base-pairing are connected with arc curves on top of the alignments. Potential CsrA-binding sites are highlighted by green shadows in the right panel

Genome-wide TSS analyses allow the identification of sRNAs, which frequently function as post-transcriptional regulators [86]. In addition to the conserved bacterial ncRNAs, RNase P RNA, SRP RNA, tmRNA, rRNAs, and 6S RNA, we identified 127 sRNAs from the chromosome and 11 from the plasmid pTE101. Remarkably, in both salinities, *~*87% of all primary reads were associated with nTSSs, and most of these reads originated from an intergenic region of only 404 bp containing three nTSSs (Fig. 5a). We identified three sRNAs originating from this genomic region and named them Aln1a, Aln1b and Aln1c for *Al* *teromo* *n* *as* sRNA 1a-c. These sRNAs share 99–96% nucleotide identity among *A. macleodii* and 77% identity, on average, to homologous sequences in the 11 *A. mediterranea* strains (Fig. 5b). A previous RNA-seq study revealed the high expression of this region, comparable to ribosomal genes, in *A. mediterranea* [33]. To check expression of the Aln1 sRNAs in situ, we mapped an existing marine metatranscriptomic data set to the genome of *A. macleodii* Te101. In this data set, which tracked biomass demise of oceanic *Trichodesmium* populations [19], we detected high expression and upregulation of these three sRNAs. Hence, we conclude that Aln1a-c and their analogs must play a conserved role in some copiotrophic bacteria, at least across the genus level of *Alteromonas*.

Aln1a-c share a similar secondary structure with a high frequency of A(N)GGA motifs in the single-stranded regions, which is the typical binding motif of the CsrA protein [48, 87] (Fig. 5b). Homologs of the enterobacterial CsrA exist in other bacteria, where they are also called repressor of stationary phase metabolites (Rsm) or catabolite repression control (Crc) in *Pseudomonas* [88, 89]. These proteins are sequence-specific RNA-binding proteins that alter the translation and/or stability of mRNA targets [90, 91] and are themselves controlled by binding to antagonistic sRNAs of the RsmX-Z/CsrB-C family [92, 93] detected in a wide range of bacteria, including *Enterobacteriaceae*, *Legionella pneumophila*, *Erwinia*, *Vibrio*, and *Pseudomonas* [94–99]. RsmX-Z/CsrB-C sRNAs share the occurrence of multiple binding sites for CsrA and Rsm, respectively, with which they can sequester and antagonize the respective regulatory proteins. Notably, *Alteromonas* Te101 possesses a CsrA (TE101_05290) homolog, as shown by the 87% sequence identify with the *E. coli* homolog and the presence of likely autoregulatory A(N)GGA motifs in its ribosome binding region. We conclude that Aln1a-c, together with the protein Te101_05290, constitute the *Alteromonas* homolog of the CsrA-B-C system. Moreover, whereas Aln1a was driven by the most active promoter under both conditions, the additional activation of Aln1b and Aln1c transcription at 43 ppt (Fig. 5a and Table S4) suggests that this system plays an important role in the regulation of copiotrophic lifestyle decisions.

We subsequently estimated the potential targets of the *Alteromonas* Te101 CsrA-Aln1a-c system (Table S13). The availability of the precise 5′-UTR sequences was advantageous for this analysis, and 126 potential CsrA target genes were predicted. To include potential targets without an associated 5′-UTR, we applied the CSRA_TARGET algorithm to scan the intergenic regions and 5′ ends of genes, yielding 35 additional targets (Table S13). Consistent with recent findings in *Legionella pneumophila* [100], both *rpoS* and *csrA* may be repressed by CsrA in *Alteromonas* Te101 by blocking the respective ribosome binding sites (Fig. 5a). In addition, multiple genes associated with ribosomes, flagella, the cell cycle and DNA replication, transport and receptor functions were predicted to be regulated by CsrA (Table S13).

**Fig. 6:**
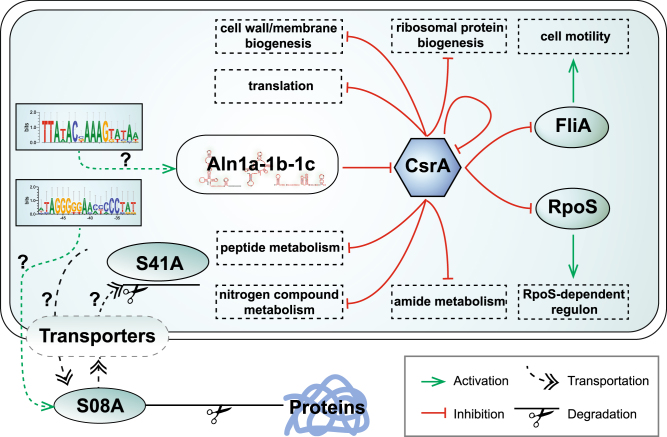
Proposed regulatory mechanisms in *Alteromonas* Te101 during transition to copiotrophic growth. Aln1 sRNA transcription becomes stimulated at 43 ppt. The additional Aln1 sRNAs sequester CsrA. Thus, the target mRNAs translationally repressed by CsrA become relieved. Key regulatory proteins such as RpoS become translated and activate a downstream cascade of events. Hence, the RpoS regulon is activated under nutrient-rich conditions, contrary to the situation in enterobacteria where RpoS is associated with stressful conditions including nutrient deprivation. Five of the predicted CsrA targets share the presence of a sequence motif within their promoters with the Aln1a gene (Figure S6), which may mediate their concerted regulation through an unknown regulator in a feed-forward manner. The stimulated transcription of peptidases and transport systems sustains the hydrolysis and uptake of external proteins. Question marks represent hypothesized or unknown regulatory mechanisms

A conserved motif has been identified in the promoter region of Aln1a (Figure S6). This motif might be involved in regulating the transcription of Aln1a and of other genes, including two TonB-dependent receptors (TE101_02175 and TE101_05420), two chemotaxis proteins (TE101_13385 and TE101_14825), porin (TE101_06395), pilus biogenesis protein (TE101_14740), ABC transporter substrate-binding protein (TE101_03865), AI-2E family transporter (TE101_15860), MFS transporter (TE101_09350), and a diguanylate cyclase (TE101_07475). Intriguingly, five of the CsrA target genes also possess the Aln1a promoter motif (Figure S6), including the type IV pilus assembly protein PilA (TE101_14740), the AI-2E family transporter (TE101_15860), the type IV secretion protein Rhs (TE101_16665), and the uncharacterized hypothetical proteins TE101_16690 and TE101_09190, indicating that these genes might be co-regulated by the same transcription factor and co-repressed by CsrA. The *csrA* gene is transcribed from three distinct TSSs, all of which were downregulated at 43 ppt (Fig. 5a). Hence, our results point to a central function of the CsrA-Aln1a-c system in the control of copiotrophic growth when organic matter becomes available (Fig. 6). Both the lowered transcription of *csrA* and the activated transcription of Aln1 sRNAs led to less available CsrA. Thus, the CsrA-mediated repression of translation becomes relieved, thereby stimulating peptide and nitrogen metabolism, ribosome biosynthesis and translation, membrane and cell wall biogenesis.

In addition to fundamental physiologic processes, these events can affect cellular behavior, that is, motility. Previous studies revealed that CsrA regulates biofilm formation [101], activates the expression of the flagellar operon *flhDC* in *E. coli* [92], regulates virulence factors [102], and impacts carbon metabolism and quorum sensing [103, 104]. Therefore, the predicted targets and observed regulatory changes linked to the *Alteromonas* Te101 CsrA-Aln1 system resemble the functions of the CsrA-B-C system in pathogenic bacteria. However, in its own ecological context, the CsrA-Aln1 system provides *Alteromonas* with a one-for-all solution to transiently activate all downstream pathways to benefit from nutrient hot-spots appearing in its environment.

## Conclusions

The first primary transcriptome of an opportunistic marine bacterium responding to lysing phytoplankton is presented. Our data suggest the different pathways by which copiotrophs can benefit during stochastic inputs of organic matter that appear in an otherwise nutrient-poor oligotrophic environment. The assembly and growth of the flagellar filament, protein synthesis, proteolysis, and modification of the structure of the phospholipid bilayer were all significantly upregulated under our 43 ppt experimental condition, which impaired growth of the otherwise dominant cyanobacteria *Trichodesmium*. Our transcriptome data further indicate that *Alteromonas* secretes extracellular peptidases such as S08A to facilitate the assimilation of released nutrients. Single-nucleotide resolution promoter mapping allowed the identification of complex regulatory mechanisms that are involved in these processes. Major upregulated sigma factors were RpoS (sigma S) and FliA (sigma 28). *Alteromonas* employs CsrA and the three CsrA-sequestering sRNAs, Aln1a-c, as a system for post-transcriptionally regulating the expression of up to 161 genes. We conclude that the CsrA-Aln1a-c system constitutes a major mechanism involved in the integration of copiotrophic growth when transient nutrient hotspots appear, possibly guiding cellular decisions between the metabolically expensive, yet competitive motile and the inexpensive, non-motile lifestyle.

## Electronic supplementary material

Supplemental Information

Dataset with Tables S1 to S13
