# Supplementary material for: Benefit from decline: the primary transcriptome of Alteromonas macleodii str. Te101 during Trichodesmium demise
Source: ISME J. 2018 Jan 15;12(4):981–96. doi: 10.1038/s41396-017-0034-4 (PMC5864184; doi:10.1038/s41396-017-0034-4)
Supplement: Supplementary file 1 — Supplemental Information [file 41396_2017_34_MOESM1_ESM.pdf]

Benefit from decline: The primary transcriptome of *Alteromonas macleodii* str. Te101 during *Trichodesmium* demise

Shengwei Hou[1,$], Mario López-Pérez[2,$], Ulrike Pfreundt[1,3], Natalia Belkin[4], Kurt Stüber[5], Bruno Huettel[5], Richard Reinhardt[5], Ilana Berman-Frank[4], Francisco Rodriguez-Valera[2], Wolfgang R. Hess[1,*]

Supplementary Information

Supplementary methods

Library preparation, read cleaning and taxonomic classification

Details of the RNA isolation and sampling were described previously (Pfreundt *et al.*, 2014; Pade *et al.*, 2016). Primary transcriptomes were inferred by the genome-wide mapping of transcription start sites (TSSs). For this aim, differential RNA-Seq (dRNA-Seq) (Sharma *et al.*, 2010) was used, in which the primary transcripts resulting from the initiation of transcription are selectively sequenced. This approach relies on the 5'P-dependent terminator exonuclease (TEX) activity, which specifically degrades processed transcripts while primary transcripts with their 5'triphosphates are kept intact. Recently, we have extended this approach to the microbial community sampled from the Red Sea and identified the suite of active TSSs from five different organisms representing all three domains of life, showing the potential of this approach in a complex microbial community context (Hou *et al.*, 2016).

For read cleaning and quality control we followed the workflow of Hou *et al.* (2016). Raw reads were checked with FastQC v0.10.1 (Andrews, 2010), adapters were removed with Cutadapt v1.0 (Martin, 2011), low quality reads were trimmed or removed with fastq_quality_trimmer from the FASTX-Toolkit v0.0.13 (available at http://hannonlab.cshl.edu/fastx_toolkit/), the remaining high quality reads were converted to fasta format, clustered if identical and rRNA reads removed using SortMeRNA v1.9 (Kopylova *et al.*, 2012). The taxonomic classification of the non-rRNA reads was obtained using Centrifuge v1.0.3 (Kim *et al.*, 2016) with default parameters (database updated on Dec. 6th, 2016). The results were imported into Pavian v0.1.3 (Breitwieser and Salzberg, 2016) for visualization with a minimum score of 1,000 and minimum length of 60.

Prediction of TSSs

For the bioinformatic analysis of dRNA-Seq data and TSS prediction we applied a replicate-assisted background subtraction algorithm. Reads from all libraries were aligned to the

Alteromonas Te101 genome at 99% identity using segemehl v0.2.0 (Hoffmann *et al.*, 2009), alignments were then converted to Artemis (Rutherford *et al.*, 2000) compatible tabular files (GRP format) with genome coordinates, number of reads starting at each nucleotide position ("number of reads starting", NRS), and per-nucleotide-coverage using GRPutils (https://github.com/housw/GRPutils). For each salinity condition, the GRP files of the dRNA-Seq libraries and the minus-libraries were normalized against the largest library based on the sum of NRS values. To eliminate the noise from putative RNA-processing sites, the square-root-scaled NRS values of the minus-libraries were subtracted from the NRS values of the corresponding dRNA-Seq libraries yielding subtracted NRS values. The core TSSs was defined separately for each condition at the nucleotide positions of read starts after background subtraction. To account for the multiple tightly spaced starts observed at some sites, a window of 5 nt up- and 5 nt downstream of the core TSS was considered, the aggregated NRS values calculated as the sum of NRS in this window, while the local maximum defined the TSS. To account for TSSs among different conditions, the core TSS defined separately for each condition was compared, overlapping TSSs integrated and the maximum value was taken for each TSS among different conditions. This produced a pseudo-count for each core TSS summarizing information from different libraries, biological replicates and conditions. The core TSS were classified into gTSS, iTSS, aTSS and nTSS based on the genomic context, and a quality filter of pseudoCount ≥10, covRatio ≥0.5, locCovEnrich ≥0.5 and locTssEnrich ≥0.3 to define the precise TSS positions. A TSS located within 200 nt upstream of an annotated protein-coding gene, or giving rise to reads overlapping such a gene, was classified as a gene TSS (gTSS). TSS located within an annotated gene or antisense to it (plus 50 nt up- or downstream) were called iTSS and aTSS, respectively. TSS located in intergenic regions or upstream of an ncRNA including rRNA and tRNA genes were designated as non-coding TSS (nTSS). Finally, the original alignments were mapped and aggregated to these *bona fide* TSS positions to get the

raw read counts initiated from these positions. The implementation of this algorithm can be found at https://github.com/housw/GRPutils/blob/master/tss_analysis_pipline.sh.

Differential expression analysis

For differential expression analysis, the TSS count table obtained from the TSS prediction was filtered for lowly expressed TSS (<10 reads from the 4 libraries combined) and TSS associated with rRNAs and tRNAs and then normalized using the Trimmed Mean of M-values (TMM) method in edgeR v3.14.0 (Robinson *et al.*, 2010). Dispersions were estimated by treating samples from 37 ppt and 43 ppt as replicates using the quantile-adjusted conditional maximum likelihood method (qCML) and differentially expressed TSS between 43 ppt and 37 ppt were called using the exactTest function in edgeR with an adjusted p-value cutoff of 0.05.

To characterize the functions of genes up-regulated at 43 ppt, GO terms were annotated for the whole proteome using Blast2GO v4.0.7 (Conesa *et al.*, 2005). All the determined differentially expressed TSSs were selected, then the genes driven by gTSS, the genes downstream of nTSS, iTSS and aTSS within 1 kb were extracted as the query gene set. Enriched GO terms were determined using hypergeometric tests implemented in GOstats v2.38.1 (Falcon and Gentleman, 2007) with the GO terms of the whole proteome as background. The multiple test corrections were performed using qvalue v2.4.2 (available at http://github.com/jdstorey/qvalue) in R. Enriched GO terms were semantically clustered using the REVIGO (Supek *et al.*, 2011) online server.

CsrA target prediction and motif finding

Based on predicted gTSS positions, all 5'UTRs were extracted and checked for potential CsrA binding sites using the regular expression "GGA[ACGT]{4,70}GGA[ACGT]{2,12}$", similar to the one used in a previous study (Naghdi *et al.*, 2017), which looks for a GGA motif 4 to 70 nt upstream of the Shine-Dalgarno (SD) sequence within 5'UTRs. Genes without SD sequences

5 to 15 nt upstream of translation start sites were considered as targets when ≥3 "GGA" were found in their 5'UTR and the normalized frequency of "GGA" per 100 nt was ≥3. To include genes without gTSSs, we also applied the CSRA_TARGET (Kulkarni *et al.*, 2014) algorithm to genome-wide scan intergenic regions 300 nt upstream of and 50 nt following the ORF start. To detect regulatory motifs in promoter regions, sequences 200 nt upstream of predicted TSSs were extracted and submitted to the XXmotif web server (Luehr *et al.*, 2012) with default parameters except no masking, the E-value cutoff for trusted identified motifs was set to 0.001.

Supplementary Figures

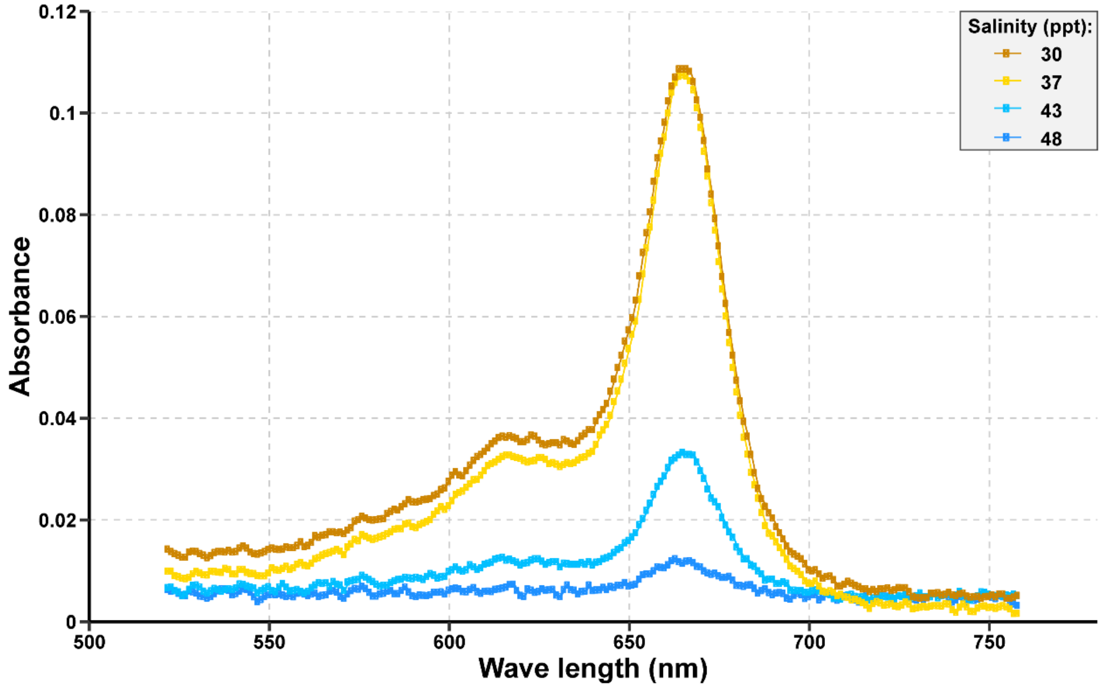

Figure S1. Spectral scan of pigment absorbance of the *Trichodesmium* cultures on day 9. Cells were collected on GFF and pigments were extracted using 90% boiling methanol (de Marsac and Houmard, 1988). Pigment absorbance scans were analyzed with Cary 300 spectrophotometer (Agilent Technologies) between 520-760 nm in intervals of 1 nm.

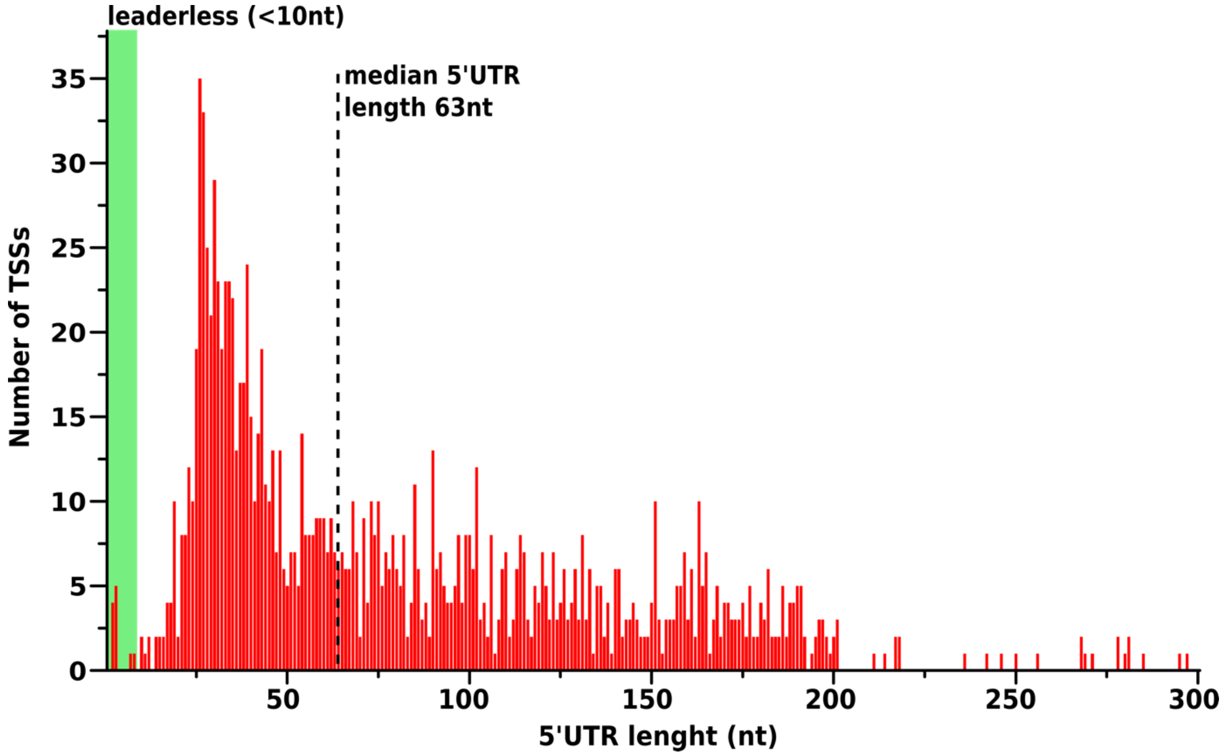

Figure S2. Length distribution of 5’ UTRs in *Alteromonas* Te101.

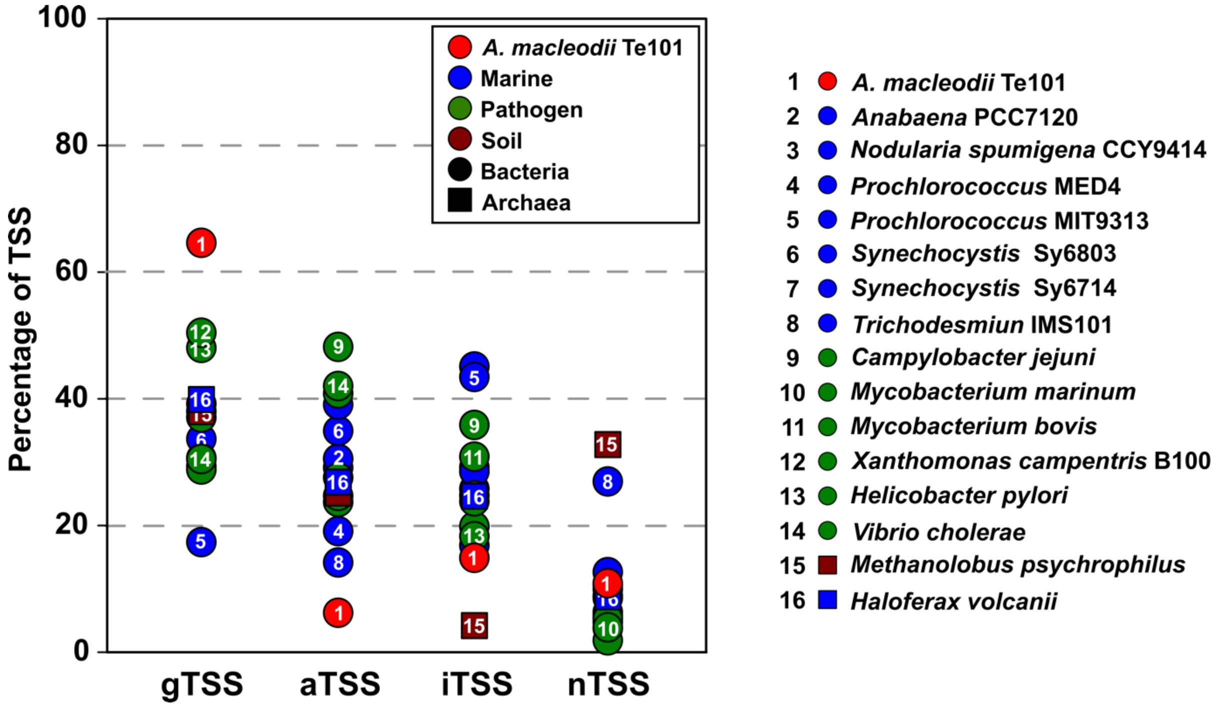

Figure S3. Percentage of the different TSS fractions predicted by several published dRNA-seq data of other bacteria and archaea in different environments.

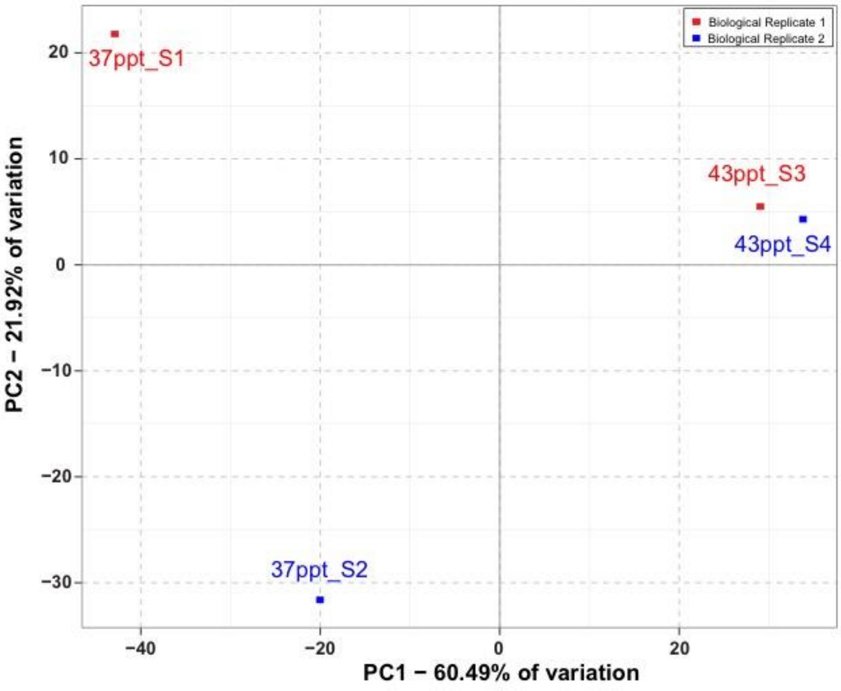

Figure S4. Principal component analysis of *Alteromonas* Te101 transcriptome based on logarithm transformed normalized count data.

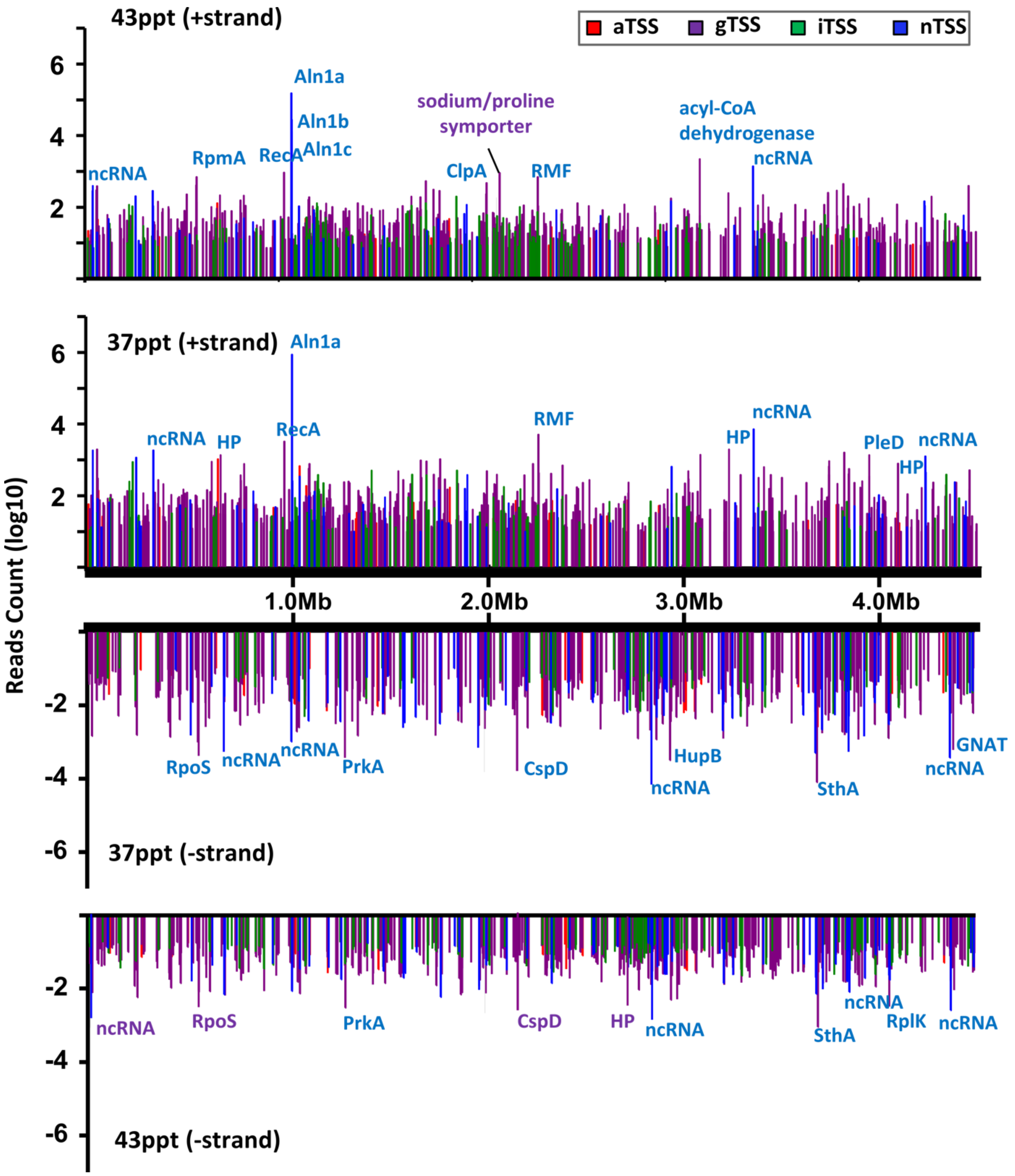

Figure S5. Genome-wide distribution of TSS in *Alteromonas* Te101.

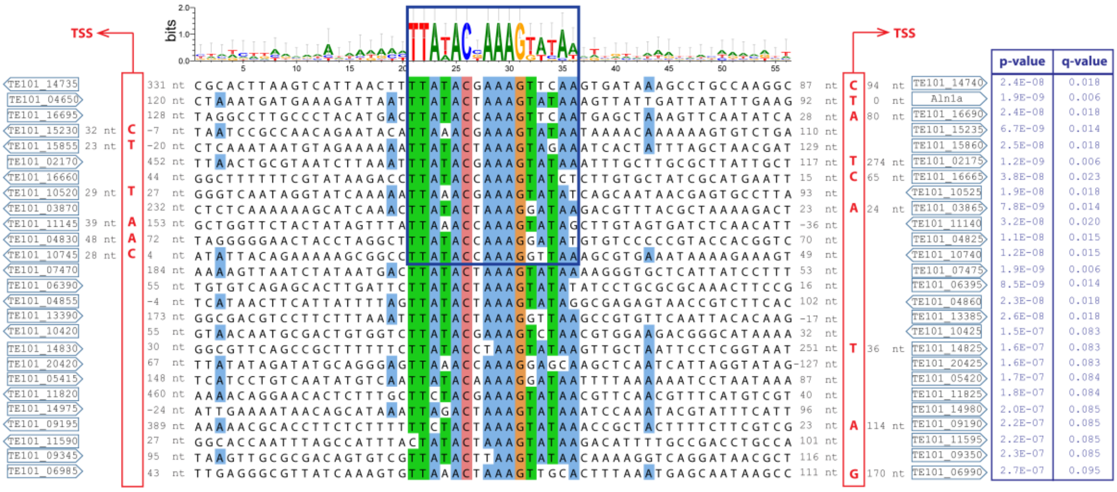

Figure S6. Identified promoter motifs upstream of Aln1a and other genes. The identified motif and flanking regions were shown in the middle, sequence alignment was shaded with an identity cutoff of 40%. Proposed associated TSSs were shown in bold and red font. Numbers show the distances between gene start/stop codons and associated TSSs, or the flanking regions when no TSSs were identified. The sequences within the dark blue square were used to generate the motif profile, which was further used to scan the *A. macleodii* Te101 genome to detect the other occurrences using FIMO (Grant *et al.*, 2011) from MEME Suite v4.12.0 (Bailey *et al.*, 2015). The probabilities and adjusted q-values of each occurrence were shown in the right columns. The identified motif was visualized using WebLogo v3.1 online server (Crooks *et al.*, 2004).

Supplementary Tables

Table S1. Identified flexible genomic islands of *Alteromonas macleodii* str. Te101 genome.

Table S2. Detected *Alteromonas* TSSs associated with leaderless transcripts and 5′-UTRs ≥200 nt.

Table S3. All *Alteromonas* TSSs identified in this work.

Table S4. All *Alteromonas* TSSs up-regulated at 43 ppt.

Table S5. All *Alteromonas* TSSs down-regulated at 43 ppt.

Table S6. Enriched GO terms of all up-regulated genes at 43 ppt.

Table S7. Detected TSSs involved in the motility gene cluster.

Table S8. TSS-based differential expression analysis of Trichodesmium erythraeum IMS101.

Table S9. Overrepresented GO terms of down-regulated Trichodesmium genes at 43 ppt.

Table S10. Subfamily distribution of MEROPS peptidases.

Table S11. Predicted motifs in the promoter regions of all identified TSSs.

Table S12. Relative abundance of carbohydrate active enzymes (CAZy).

Table S13. Predicted CsrA targets.

Supplementary Dataset

Supplementary Dataset 1. Genome-wide visualization of predicted TSS and coverage for the genome of *Alteromonas* Te101.

Supplementary Dataset 2. Genome-wide GO assignments of *Alteromonas* Te101 proteins.

Supplementary dataset can be accessed at https://figshare.com/s/542f9a680bd4d4c92af7.

Supplementary references

Andrews S. (2010). FastQC A quality control tool for high throughput sequence data. http://www.bioinformatics.babraham.ac.uk/projects/fastqc/ (Accessed July 14, 2014).

Bailey TL, Johnson J, Grant CE, Noble WS. (2015). The MEME Suite. *Nucleic Acids Res* **43**: W39–W49.

Breitwieser FP, Salzberg SL. (2016). Pavian: Interactive analysis of metagenomics data for microbiomics and pathogen identification. *bioRxiv* 84715.

Conesa A, Götz S, García-Gómez JM, Terol J, Talón M, Robles M. (2005). Blast2GO: a universal tool for annotation, visualization and analysis in functional genomics research. *Bioinforma Oxf Engl* **21**: 3674–3676.

Crooks GE, Hon G, Chandonia J-M, Brenner SE. (2004). WebLogo: a sequence logo generator. *Genome Res* **14**: 1188–1190.

Falcon S, Gentleman R. (2007). Using GOstats to test gene lists for GO term association. *Bioinformatics* **23**: 257–258.

Grant CE, Bailey TL, Noble WS. (2011). FIMO: scanning for occurrences of a given motif. *Bioinformatics* **27**: 1017–1018.

Hoffmann S, Otto C, Kurtz S, Sharma CM, Khaitovich P, Vogel J, *et al.* (2009). Fast mapping of short sequences with mismatches, insertions and deletions using index structures. *PLoS Comput Biol* **5**: e1000502.

Hou S, Pfreundt U, Miller D, Berman-Frank I, Hess WR. (2016). mdRNA-Seq analysis of marine microbial communities from the northern Red Sea. *Sci Rep* **6**: 35470.

Kim D, Song L, Breitwieser FP, Salzberg SL. (2016). Centrifuge: rapid and sensitive classification of metagenomic sequences. *Genome Res* **26**: 1721–1729.

Kopylova E, Noe L, Touzet H. (2012). SortMeRNA: fast and accurate filtering of ribosomal RNAs in metatranscriptomic data. *Bioinformatics* **28**: 3211–3217.

Kulkarni PR, Jia T, Kuehne SA, Kerkering TM, Morris ER, Searle MS, *et al.* (2014). A sequence-based approach for prediction of CsrA/RsmA targets in bacteria with experimental validation in *Pseudomonas aeruginosa*. *Nucleic Acids Res* **42**: 6811–6825.

Luehr S, Hartmann H, Söding J. (2012). The XXmotif web server for eXhaustive, weight matriX-based motif discovery in nucleotide sequences. *Nucleic Acids Res* **40**: W104-109.

de Marsac NT, Houmard J. (1988). Complementary chromatic adaptation: Physiological conditions and action spectra. In: Cyanobacteria Vol. 167. *Methods in Enzymology*. Academic Press, pp 318–328.

Martin M. (2011). Cutadapt removes adapter sequences from high-throughput sequencing reads. *EMBnet.journal* **17**: 10.

Naghdi MR, Smail K, Wang JX, Wade F, Breaker RR, Perreault J. (2017). Search for 5'-leader regulatory RNA structures based on gene annotation aided by the RiboGap database. *Methods San Diego Calif* **117**: 3–13.

Pade N, Michalik D, Ruth W, Belkin N, Hess WR, Berman-Frank I, *et al.* (2016). Trimethylated homoserine functions as the major compatible solute in the globally significant oceanic cyanobacterium *Trichodesmium*. *Proc Natl Acad Sci U S A* **113**: 13191–13196.

Pfreundt U, Kopf M, Belkin N, Berman-Frank I, Hess WR. (2014). The primary transcriptome of the marine diazotroph *Trichodesmium erythraeum* IMS101. *Sci Rep* **4**: 6187.

Robinson MD, McCarthy DJ, Smyth GK. (2010). edgeR: a Bioconductor package for differential expression analysis of digital gene expression data. *Bioinformatics* **26**: 139–140.

Rutherford K, Parkhill J, Crook J, Horsnell T, Rice P, Rajandream M-A, *et al.* (2000). Artemis: sequence visualization and annotation. *Bioinformatics* **16**: 944–945.

Sharma CM, Hoffmann S, Darfeuille F, Reignier J, Findeiss S, Sittka A, *et al.* (2010). The primary transcriptome of the major human pathogen *Helicobacter pylori*. *Nature* **464**: 250–255.

Supek F, Bošnjak M, Škunca N, Šmuc T. (2011). REVIGO summarizes and visualizes long lists of gene ontology terms. *PloS One* **6**: e21800.
